# Reduced Global-Brain Functional Connectivity and Its Relationship With Symptomatic Severity in Cervical Dystonia

**DOI:** 10.3389/fneur.2019.01358

**Published:** 2020-01-10

**Authors:** Pan Pan, Shubao Wei, Yangpan Ou, Wenyan Jiang, Wenmei Li, Yiwu Lei, Feng Liu, Wenbin Guo, Shuguang Luo

**Affiliations:** ^1^Department of Psychiatry, The Second Xiangya Hospital of Central South University, Changsha, China; ^2^National Clinical Research Center on Mental Disorders, Changsha, China; ^3^Department of Neurology, The First Affiliated Hospital of Guangxi Medical University, Nanning, China; ^4^Department of Radiology, The First Affiliated Hospital of Guangxi Medical University, Nanning, China; ^5^Department of Radiology, Tianjin Medical University General Hospital, Tianjin, China

**Keywords:** cervical dystonia, global-brain functional connectivity, precentral gyrus, supplementary motor area, network

## Abstract

**Background:** Altered functional connectivity (FC) is related to pathophysiology of patients with cervical dystonia (CD). However, inconsistent results may be obtained due to different selected regions of interest. We explored voxel-wise brain-wide FC changes in patients with CD at rest in an unbiased manner and analyzed their correlations with symptomatic severity using the Tsui scale.

**Method:** A total of 19 patients with CD and 21 sex- and age-matched healthy controls underwent resting-state functional magnetic resonance imaging scans. Global-brain FC (GFC) was applied to analyze the images. Support vector machine was used to distinguish the patients from the controls.

**Results:** Patients with CD exhibited decreased GFC in the right precentral gyrus and right supplementary motor area (SMA) that belonged to the M1-SMA motor network. Significantly negative correlation was observed between GFC values in the right precentral gyrus and symptomatic severity in the patients (*r* = −0.476, *p* = 0.039, uncorrected). Decreased GFC values in these two brain regions could be utilized to differentiate the patients from the controls with good accuracies, sensitivities and specificities (83.33, 85.71, and 80.95% in the right precentral gyrus; and 87.59, 89.49, and 85.71% in the right SMA).

**Conclusions:** Our investigation suggests that patients with CD show reduced GFC in brain regions of the M1-SMA motor network and provides further insights into the pathophysiology of CD. GFC values in the right precentral gyrus and right SMA may be used as potential biomarkers to recognize the patients from the controls.

## Introduction

Cervical dystonia (CD), known as spasmodic torticollis, is the most common type of focal dystonia with estimated prevalence of 28–183 cases per million individuals (1, 2). CD is caused by abnormal impulse of central nervous system (CNS) resulting in cervical muscle group paroxysmal involuntary contraction, and thus presents abnormal posture of head and neck. Simple rotatory torticollis is the most common type occurring in >50% of cases (3). Other types can be classified into laterocollis, anterocollis, retrocollos, or tremor (4). Patients with CD are often accompanied with neck or shoulder pain and tremor (5, 6). The chronic neck pain caused by CD leads to disability or low quality of life. Thus, it is meaningful to accurately identify patients with CD and provide them with effective treatment in clinical trials. However, no curative treatment is available at present, and the pathophysiology underlying the disorder remains poorly understood.

Rapid advances in neuroimaging techniques suggest that CD is not a disease with abnormality in an isolated brain region but a chronic disorder involving damage in multiple brain networks (7). Patients with CD showed structural alterations in lentiform nucleus, basal ganglia (8, 9), internal globus pallidus (10), thalamus, cerebellum, motor cortex, and supplementary motor area (SMA), putamen, right visual cortex, and right dorsal lateral prefrontal cortex (11). Altered functional connectivity (FC) was observed in brain regions including premotor cortex, prefrontal cortex, parietal cortex, middle temporal gyrus, SMA, primary motor area (M1), secondary somatosensory cortex, right supramarginal gyrus, and a network that comprised anterior cingulate cortex (12–16).

However, findings of the above-mentioned studies are inconsistent in terms of special brain regions. For example, conflicting results of an enhancement in gray matter volume in the motor cortex and SMA but a reduction in gray matter were reported in the same areas (11). Several analyses on regions of interest (ROI) revealed alterations in cerebellar gray matter volume (11), but other researchers found no such abnormality in patients with CD (13). Patients with CD displayed increased connectivity in the premotor, prefrontal cortex, and parietal cortex but had decreased paradox connectivity in the same regions (7, 13). The inconsistency may be partly due to a seed-based FC method (ROI) or an independent component analysis approach used to explore the brain mechanisms in patients with CD. These approach are useful in testing hypotheses regarding specific regions or networks but do not provide a comprehensive method for examining connectivity outside of the predetermine areas (17, 18).

Given this background, a voxel-wise global-brain FC (GFC) approach was utilized to examine the difference in large-scale functional organization in patients with CD. GFC uses a metric that does not require a priori seed or network selection and provides a measure of the connectivity of all voxels in the brain relative to all other voxels (19–24). GFC has been proven to be a powerful and replicable data-driven analysis for the identification of major intrinsic networks (18, 25). The goals of this GFC study conducted in patients with CD included the following: (1) to explore GFC differences between patients with CD and healthy controls; (2) to probe relationship between altered FC and clinical measurements in patients with CD; and (3) to examine whether GFC values in relevant brain areas may be considered potential image biomarkers in differentiating patients from healthy controls using support vector machine (SVM).

## Materials and Methods

### Subjects

A total of 21 right-handed patients with CD were referred from the First Affiliated Hospital of Guangxi Medical University. CD was diagnosed based on criteria of the dystonia diagnostic and treatment guidelines of Chinese Medical Association of neurology branch of Parkinson's disease and movement disorders group. A total of 21 right-handed healthy controls without symptoms of neurologic diseases were recruited by advertisements from local community at the same time. All participants aged from 18 to 60 years old, and healthy controls were group-matched with the patients in terms of age and sex ratio.

Patients with CD shared the following exclusion criteria: (1) consistent with diagnosis of primary CD with rotatory torticollis but obvious dystonia existed in other parts of the body except the cervical region, (2) any other neurological with the exception of dystonia, (3) other causes of secondary spasmodic torticollis that are definitely diagnosed, (4) history of related medical treatment or operation therapy within 3 months before the treatment such as Botulinum-A toxin injection (26, 27), and (5) any history of serious medical or neurological illness. The exclusion criteria for healthy controls were as follows: (1) any history of severe neuropsychiatric diseases, (2) any history of serious surgery or internal medicine diseases, and (3) any family history of severe neurological disorders in their first-degree relatives. The participants that did not reach the standard for MRI or showed alterations under conventional MRI scans were also excluded.

All patients were assessed with the Tsui scale (28) to measure symptomatic severity of CD. The study was approved by the Local Ethics Committee of the First Affiliated Hospital of Guangxi Medical University. All participants provided a written informed consent prior to the experiment.

### Image Acquisition and Preprocessing

A Siemens 3.0 T scanner was used to capture resting-state scans. All participants were required to lie still, close their eyes, and stay awake. The participants used soft earplugs and foam pads to reduce the scanning noise and head motion. The acquisition slice-order type was ascend with the following parameters: repetition time/echo time = 2,000 ms/30 ms, inversion time = 900 ms, 30 slices, 64 × 64 matrix, 90° flip angle, 240 mm field of view, 4 mm slice thickness, 0.4 mm gap, and 250 volumes lasting for 500 s. After scanning, participants were asked whether they fell asleep during the fMRI scanning, and all participants confirmed wakefulness.

Functional image data were preprocessed automatically using the DPABI software (29). The first 10 volumes were removed to ensure a steady-state condition. The fMRI time series was first corrected for within-scan acquisition time differences between slices and head motion. We excluded the participants whose head movement exceeded 2 mm of translation or 2° of rotation in any directions. All realigned images were spatially normalized to the Montreal Neurological Institute EPI template in SPM8 and resampled to 3 mm × 3 mm × 3 mm voxels (30). After normalization, the images were smoothed with a 4 mm full width on the half-maximum Gaussian kernel. The time series were further band-pass filtered (0.01–0.08 Hz) and linearly detrended. Afterward, several covariates, including Friston-24 head motion parameters acquired by rigid body correction, signal from a region centered in white matter, and signal from cerebrospinal fluid were removed. Global signal was not removed as indicated in a previous study (31). The frame-wise displacement (FD) value for each participant was calculated according to a previous study (32). Scrubbing (removing time points with FD > 0.2 mm) was also used to control the effect of head motion.

### GFC Analysis

The GFC method was similar to that used in our previous study (24). For each participant, we calculated average values of correlations between each voxel's time series and every other voxel in gray matter of the whole brain in MATLAB, which was defined as GFC of this voxel (18). The threshold setting classified voxel with probability of >0.2 as gray matter, and the gray matter mask would be produced by the gray matter probability map in SPM8 (33). The GFC values were converted into Fisher z-scores (21, 24, 34). The GFC maps were generated by combining GFC of all voxels. Thereafter, two-sample *t*-tests were conducted on the GFC maps between patients with CD and controls after the normality of the data being checked. The mean FD and age were used as covariates of no interest to limit the possible effects of these variables. The significance level was set as *p* < 0.05 by using the family wise error (FWE) correction method.

### Correlation Analysis

We extracted mean z values from brain clusters with abnormal GFC. After checking normality of the data, Pearson correlations were performed to determine the relationship between GFC values and Tsui total scores in the patients. The significance level was set at *p* < 0.05.

### Classification Analysis by Using SVM

SVM was applied to examine whether decreased GFC in several brain regions could be used to distinguish the patients from the controls (35). The LIBSVM software adopted a “leave-one-out” (LOO) approach that was cross-validated to obtain good sensitivity and specificity. In our study, given a dataset of 19 samples, the LOO-based validation was performed with 19 iteration. In each iteration, the classifier was trained with 19-1 samples and tested on the remaining sample. The type of kernel was the default Gaussian kernel in LIBSVM (33).

We adopted a 5-fold cross-validation method to validate the SVM results. Each sample was randomly divided into five subgroups. The first 4 subgroups were taken as training sets and the fifth subgroup was taken as a test set to obtain a global accuracy. Moreover, results were validated by a permutation test, which ran 10,000 times for each sample to get a global accuracy.

## Results

### Characteristics of the Subjects

The data of 2 patients were excluded due to excessive head movement. Consequently, the final sample included 19 patients and 21 controls. Continuous variables, including age, years of education, and FD, were analyzed with two-sample *t*-tests after the normality of the data being checked. A Chi-squared test was used for sex distribution.

The differences in age (*p* = 0.75), sex ratio (*p* = 0.22), and FD (*p* = 0.51) between the patients and controls were not statistically significant. The information of demographic and clinical characteristics of the included subjects were listed in Table 1.

**Table 1:** Characteristics of participants.

**Variables**	**Patients (*n* = 19)**	**Controls (*n* = 21)**	***p*-value**
Age (years)	38.74 ± 10.71	39.62 ± 6.62	0.75^b^
Sex (male/female)	9/10	6/15	0.22^a^
FD (mm)	0.02 ± 0.02	0.03 ± 0.02	0.51^b^
Illness duration (months)	24.29 ± 31.26		
Symptom severity	16.32 ± 4.45		

a *The p-value for sex distribution was obtained by a chi-square test*.

b *The p-values were obtained by two samples t-tests*.

*FD, Framewise displacement*.

### Group Differences in GFC

**Figure 1:**
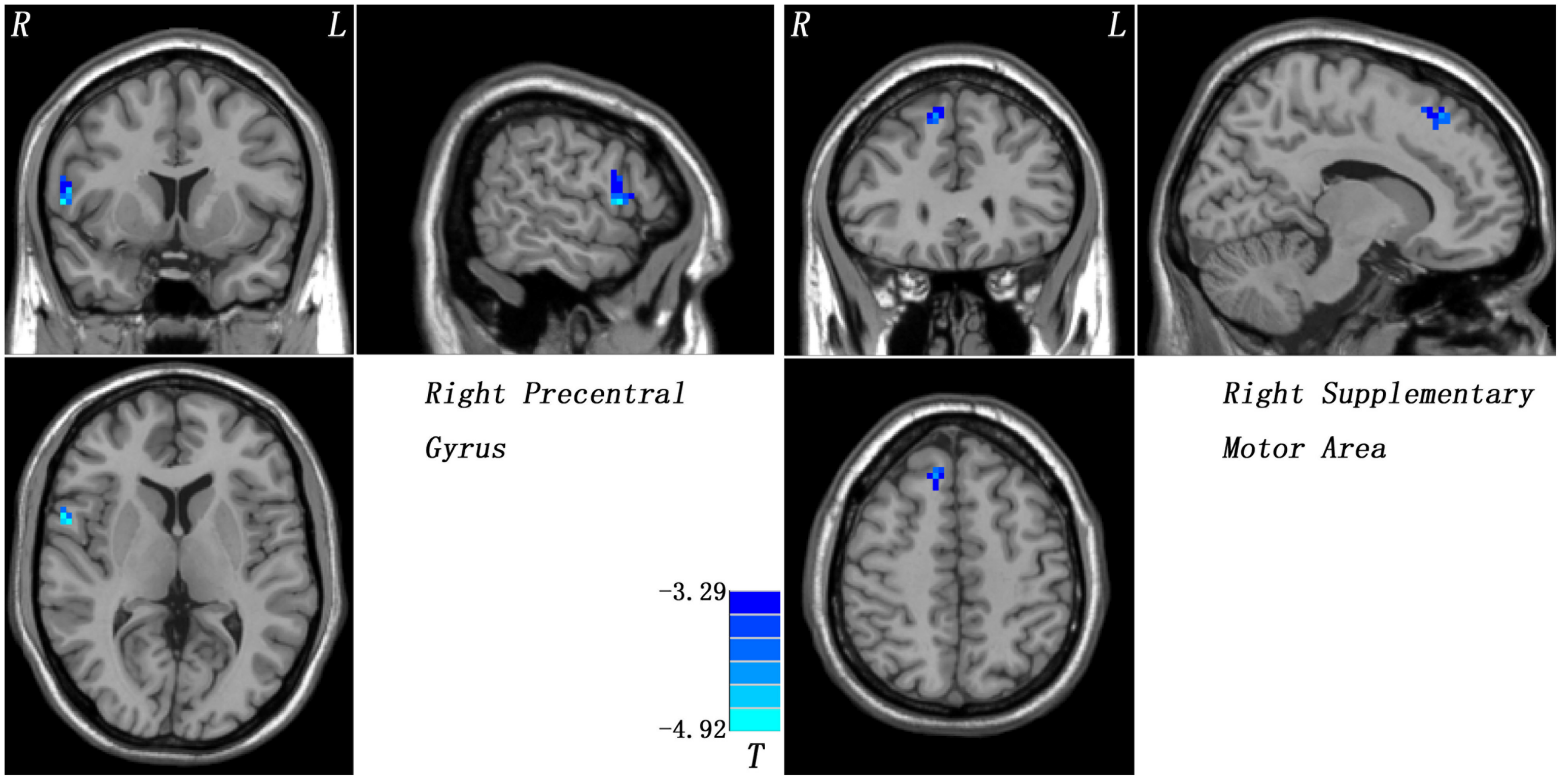
Reduced GFC in patients with cervical dystonia relative to healthy controls. GFC, global-brain functional connectivity.

Compared with the controls, patients with CD exhibited decreased GFC in the right precentral gyrus and right SMA (Figure 1 and Table 2). No brain region exhibited increased GFC in the patients relative to the controls.

**Table 2:** Regions with decreased GFC in patients.

**Cluster location**	**Peak (MNI)**			**Numbers of voxel**	***T*-value**	***p-*value**
	***x***	***y***	***z***			
Right precentral gyrus	57	6	9	19	−4.7764	<0.001
Right supplementary motor area	12	30	51	36	−4.3817	<0.001

*GFC, global-brain functional connectivity; MNI, Montreal Neurological Institute*.

### Correlations Between GFC and Clinical Variables

**Figure 2:**
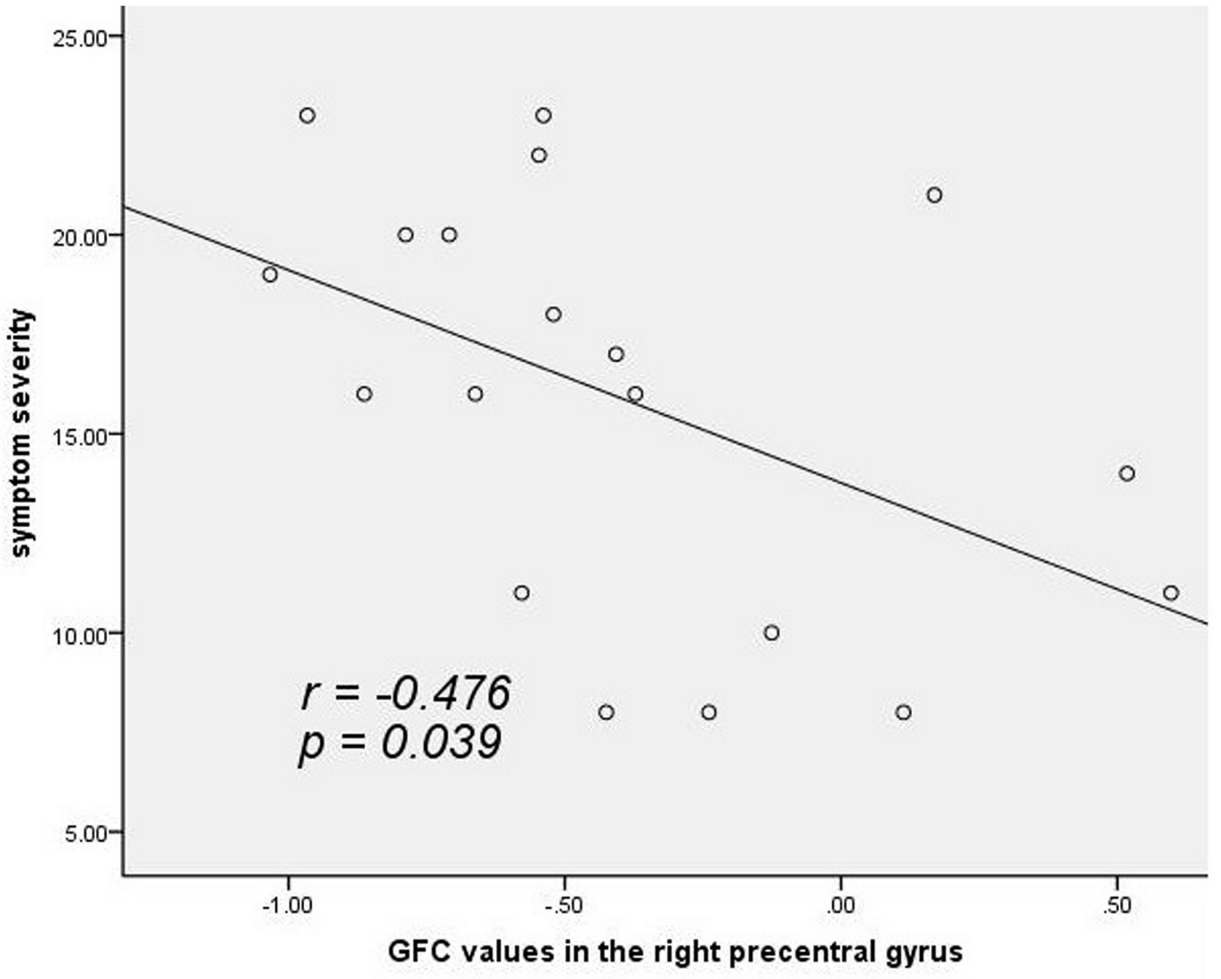
A negative correlation (*r* = −0.476, *p* = 0.039, uncorrected) between GFC values in the right precentral gyrus and symptomatic severity in patients with cervical dystonia. GFC, global-brain functional connectivity.

As shown in Figure 2, a negative correlation was observed between GFC values in the right precentral gyrus and symptomatic severity in the patients (*r* = −0.476, *p* = 0.039, uncorrected). The correlation was not significant at Bonferroni corrected *p* < 0.05/2 = 0.025 (for the two clusters).

### SVM Results

**Figure 3:**
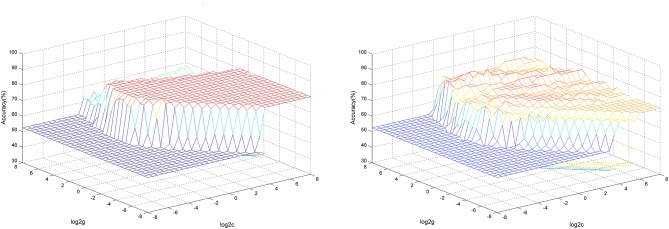
3D view of classified accuracy with the best parameters using GFC values in the right precentral gyrus and right SMA to differentiate the patients from the controls. The results were obtained in LIBSVM using a “leave-one-out” approach with default Gaussian kernel. Left: Using decreased GFC values in the right SMA to differentiate the patients from the controls. Right: Using decreased GFC values in the right precentral gyrus to differentiate the patients from the controls. SVM, support vector machine; GFC, global-brain functional connectivity; SMA, supplementary motor area.

SVM analysis was conducted to determine whether GFC values in these brain areas could distinguish patients with CD from healthy controls with good sensitivity and specificity. The decreased GFC values in two brain regions exhibited high accuracies, sensitivities and specificities (83.33, 85.71, and 80.95% in the right precentral gyrus; and 87.59, 89.49, and 85.71% in the right SMA) in differentiating patients with CD from healthy controls (Figure 3).

We used both the 5-fold cross-validation and permutation test methods to validate the SVM results. The global balanced accuracy was 80.00 and 68.42% in the right precentral gyrus and right SMA using the 5-fold cross-validation method. By contrast, the global accuracy was 0.8023 (*p* < 0.001) and 0.8071 (*p* < 0.001) in the right precentral gyrus and right SMA using the permutation test.

## Discussion

Patients with CD exhibited significantly decreased GFC values in brain regions of the M1-SMA motor network compared with healthy controls. Moreover, GFC values in the right precentral gyrus were negatively correlated with symptomatic severity in the patients. GFC values in these areas could correctly identify patients from healthy controls with good sensitivity, specificity, and accuracy.

Previous studies indicate that dystonia is a disorder of motor organization, programming, execution, and sensorimotor integration (36). The precentral gyrus (as the M1), located between the central sulcus and anterior central sulcus on the dorsolateral side of the frontal lobe, is closely related to motor preparation and execution. The main function of precentral gyrus is to convert programmed behavioral instructions from other brain regions into signals that encode various movements, such as muscle contractions, strength and duration. Precentral gyrus receives projections from postcentral gyrus and part of the secondary somatosensory cortex of the dominant hemisphere, which contain information on the contralateral derma, muscle and arthrosis, and subsequently corrects movement (37). The abnormality in this motor system may show reduced surround inhibition, resulting in unnecessary and redundant muscle contractions beyond specific behavior (12). Therefore, decreased GFC in the right precentral gyrus of patients with CD may lead to impaired movement preparation or movement inhibition, resulting in symptoms of uncontrolled muscle contraction in shoulder and neck.

GFC values in the right precentral gyrus were negatively correlated with symptomatic severity of the patients. Significantly decreased gray matter volume in the right precentral gyrus was observed in patients with cervical spondylotic myelopathy, and increased gray matter volume was found in the same area after surgery compared with baseline data (38). One possible explanation for increased gray matter volume in the motor cortex was cortical plasticity. Meanwhile, an fMRI study revealed an abnormally low cortical activity in precentral gyrus in patients with focal dystonia (39). The pathological involuntary twisting and contraction of the cervical muscles in patients with CD might increase frequency of muscle activity, which was equivalent to passive and orderly movement training. Dystonia excessive muscle spasm would cause low GFC values in the right precentral gyrus, thereby resulting in the negative correlation between GFC values in the right precentral gyrus and symptomatic severity in the patients. The negative correlation observed in the present study indicated that decreased GFC values in the right precentral gyrus could serve as a quantitative marker for evaluation of clinical symptomatic severity in the patients.

The SMA, located at the medial wall of superior frontal gyrus, is a brain region associated with voluntary movement (40). It is associated with high motor regulations, such as initiation of movement. Several clinical observations (41, 42) revealed that patients with impaired function in the SMA showed delayed movement initiation, difficulty in acting smoothly, and poorly organized movements. Functional changes observed in the SMA were associated with increasing upper extremity function scores during rehabilitation (43). Hence, decreased GFC values in the SMA may be associated with the involuntary spasm of the focal muscle in the patients.

Kasess et al. proposed a closed-loop control circuit composed of basal ganglia thalamic neurons connecting M1 and SMA to subserve motor task execution interactively (44, 45). Reciprocal interconnections between M1 and SMA were found in patients with epilepsy with focal seizures as the main clinical manifestation (46), and fMRI revealed a reduced coupling between M1 and SMA in subcortical pathology. We also examined whether there were abnormal reciprocal interconnections between M1 and SMA in patients with CD, and found decreased FC between right M1 and right SMA in patients with CD compared with healthy controls (*p* = 0.036, Table S2). Abnormal correlation between M1 and SMA in patients with CD suggests that decreased GFC in these loops may be related to limb dyskinesia in the present study.

SVM analysis exhibited that sensitivities, specificities, and accuracies of GFC values in the right precentral gyrus and right SMA in differentiating the patients from the controls were >0.8, which were good for the established diagnostic indicators (47). These results were further validated by the 5-fold cross-validation and permutation test methods. Also, GFC values from 116 brain regions of the Anatomical Automatic Labeling (AAL) templates were extracted, and SVM was conducted to examine whether GFC values of 116 brain regions could differentiate the patients from the controls with good accuracies, sensitivities and specificities. As shown in Table S1, the accuracies, sensitivities and specificities of GFC values in the right precentral gyrus and right SMA were among the highest ones. Thus, decreased GFC values in these brain regions may be utilized as potential image biomarkers to discriminate patients with CD from healthy controls.

This study has some limitations. First, all patients had minimal or absent dystonic posturing in the supine during scanning. Muscle spasms in this position lead to difficult determination of whether this condition is a secondary spasm. Therefore, the influences of sensory deception in the analysis cannot be easily eliminated. Second, data on age of onset and other relevant clinical characteristics were collected retrospectively, which might have limited the accuracy of the information. Finally, due to the small sample size, this study has insufficient capacity to subdivide patients into different groups based on head rotation.

## Conclusions

The present study indicates that reduced GFC exists in brain areas of the M1-SMA motor network in patients with CD. GFC values in the right precentral gyrus and right SMA may be used as potential biomarkers to differentiate the patients from the controls. Thus, this study provides new insights into the pathological changes of GFC in CD.

## Data Availability Statement

WG had access to all the data in the study and had final responsibility for decision to submit for publication. The data will be available upon request to WG, guowenbin76@csu.edu.cn.

## Ethics Statement

All procedures performed in studies involving human participants were in accordance with the ethical standards of the institutional and/or national research committee and with the 1964 Helsinki declaration and its later amendments or comparable ethical standards. The study was approved by the Local Ethics Committee of the First Affiliated Hospital of Guangxi Medical University.

## Informed Consent

Written informed consent was obtained from all study participants.

## Author Contributions

The manuscript was written through contributions of all authors. Among them, WG and SL provided the conception of the work. SW, PP, YO, and WJ collected the data. FL, WL, and YL were responsible for data analysis and interpretation. The manuscript was drafted by author PP and critically revised by WG. All authors have given approval to final version of the manuscript.

### Conflict of Interest

The authors declare that the research was conducted in the absence of any commercial or financial relationships that could be construed as a potential conflict of interest.
