# Supplementary material for: Reduced Global-Brain Functional Connectivity and Its Relationship With Symptomatic Severity in Cervical Dystonia
Source: Front Neurol. 2020 Jan 10;10:1358. doi: 10.3389/fneur.2019.01358 (PMC6965314; doi:10.3389/fneur.2019.01358)
Supplement: Supplementary file 1 [file Table_3.DOCX]

**supplementary material**

**Title:**

**Reduced global-brain functional connectivity and its relationship with symptomatic severity in cervical dystonia**

**Authors:**

Pan Pan^a, b^, Shubao Wei^c^, Yangpan Ou^a, b^, Feng Liu^d^, Wenyan Jiang^c^, Wenmei Li^c^, Yiwu Lei^c^, Wenbin Guo^a, b^, Shuguang Luo^c^

**Affiliation/address:**

^a^ Department of Psychiatry, The Second Xiangya Hospital of Central South University, Changsha, Hunan 410011, China.

^b^ National Clinical Research Center on Mental Disorders, Changsha, Hunan 410011, China.

^C^ Department of Neurology, The First Affiliated Hospital of Guangxi Medical University, Nanning, Guangxi 530021, China.

^d^ Department of Radiology, Tianjin Medical University General Hospital. Tianjin 300000, China.

**Corresponding authors:**

Wenbin Guo

Department of Psychiatry, The Second Xiangya Hospital of Central South University, Changsha, Hunan 410011, China.

E-mail: [guowenbin76@csu.edu.cn](mailto:guowenbin76@csu.edu.cn)

Tel.: +86 731 85360921

Shuguang Luo

Department of Neurology, The First Affiliated Hospital of Guangxi Medical University, Nanning, Guangxi 530021, China.

E-mail: robert58243@sohu.com

Tel: +86 771 5356504

Table S1. The accuracies, sensitivities and specificities of GFC values in 116 brain regions of the AAL templates to differentiate the patients from the controls.

|  | Accuracy | sensitivity | specificity |
| --- | --- | --- | --- |
| Amygdala_L | 58.52% | 38.10% | 78.94% |
| Amygdala_R | 60.15% | 57.14% | 63.16% |
| Angular_L | 42.49% | 42.86% | 42.11% |
| Angular_R | 82.08% | 90.48% | 73.68% |
| Calcarine_L | 81.83% | 95.24% | 68.42% |
| Calcarine_R | 67.04% | 76.19% | 57.89% |
| Caudate_L | 74.69% | 80.95% | 68.42% |
| Caudate_R | 69.19% | 85.74% | 52.63% |
| Cingulum_Ant_L | 68.68% | 95.24% | 42.11% |
| Cingulum_Ant_R | 73.94% | 95.24% | 52.63% |
| Cingulum_Mid_L | 69.68% | 76.19% | 63.16% |
| Cingulum_Mid_R | 84.96% | 85.71% | 84.21% |
| Cingulum_Post_L | 69.93% | 71.43% | 68.42% |
| Cingulum_Post_R | 69.93% | 71.43% | 68.42% |
| Cuneus_L | 68.93% | 90.48% | 47.37% |
| Cuneus_R | 80.2% | 76.19% | 84.21% |
| Frontal_Inf_Oper_L | 82.83% | 76.19% | 89.47% |
| Frontal_Inf_Oper_R | 91.73% | 85.71% | 97.74% |
| Frontal_Inf_Orb_L | 68.68% | 95.24% | 42.11% |
| Frontal_Inf_Orb_R | 75.19% | 71.43% | 78.95% |
| Frontal_Inf_Tri_L | 68.68% | 95.24% | 26.32% |
| Frontal_Inf_Tri_R | 55.39% | 47.62% | 63.16% |
| Frontal_Med_Orb_L | 50.0% | 0% | 100% |
| Frontal_Med_Orb_R | 50.0% | 0% | 100% |
| Frontal_Mid_L | 58.77% | 33.33% | 84.21% |
| Frontal_Mid_R | 71.8% | 85.71% | 57.89% |
| Frontal_Mid_Orb_L | 85.08% | 85.95% | 84.21% |
| Frontal_Mid_Orb_R | 85.47% | 76.19% | 94.74% |
| Frontal_Sup_L | 50.0% | 0% | 100% |
| Frontal_Sup_R | 69.42% | 80.95% | 57.89% |
| Frontal_Sup_Medial_L | 82.08% | 90.48% | 73.68% |
| Frontal_Sup_Medial_R | 65.16% | 61.90% | 68.42% |
| Frontal_Sup_Orb_L | 69.68% | 76.19% | 63.16% |
| Frontal_Sup_Orb_R | 64.14% | 80.90% | 47.37% |
| Fusiform_L | 77.75% | 76.19% | 78.95% |
| Fusiform_R | 71.43% | 80.95% | 61.90% |
| Heschl_L | 60.68% | 95.24% | 26.12% |
| Heschl_R | 63.16% | 36.84% | 89.47 |
| Hippocampus_L | 50.0% | 0% | 100% |
| Hippocampus_R | 71.06% | 47.37% | 94.74% |
| Insula_L | 75.44% | 66.67% | 84.21% |
| Insula_R | 69.17% | 85.71% | 52.63% |
| Lingual_L | 73.06% | 61.90% | 84.21% |
| Lingual_R | 50.0% | 0% | 100% |
| Occipital_Inf_L | 67.79% | 61.90% | 73.68% |
| Occipital_Inf_R | 76.57% | 95.24% | 57.89% |
| Occipital_Mid_L | 75.44% | 66.67% | 84.21% |
| Occipital_Mid_R | 82.58% | 80.95% | 84.21% |
| Occipital_Sup_L | 67.04% | 76.19% | 57.89% |
| Occipital_Sup_R | 50.0% | 0% | 100% |
| Olfactory_L | 64.66% | 71.43% | 57.89% |
| Olfactory_R | 74.44% | 85.71% | 63.16% |
| Pallidum_L | 66.79% | 80.95% | 52.63% |
| Pallidum_R | 70.18% | 66.67% | 73.68% |
| Paracentral_Lobule_L | 92.36% | 89.47% | 95.24% |
| Paracentral_Lobule_R | 70.93% | 52.38% | 89.47% |
| ParaHippocampal_L | 85.85% | 90.74% | 80.95% |
| ParaHippocampal_R | 61.78% | 76.19% | 47.37% |
| Parietal_Inf_L | 72.01% | 80.95% | 63.16% |
| Parietal_Inf_R | 50.0% | 0% | 100% |
| Parietal_Sup_L | 80.70% | 66.67% | 94.74% |
| Parietal_Sup_R | 82.58% | 80.95% | 84.21% |
| Postcentral_L | 61.53% | 80.95% | 42.10% |
| Postcentral_R | 79.2% | 95.24% | 63.16% |
| Precentral_L | 69.17% | 85.71% | 52.63% |
| Precentral_R | 83.33% | 85.71% | 80.95% |
| precuneus_L | 80.20% | 84.21% | 76.19% |
| precuneus_R | 50.0% | 0% | 100% |
| Putamen_L | 65.79% | 89.47% | 42.10% |
| Putamen_R | 50.0% | 0% | 100% |
| Rectus_L | 61.16% | 84.21% | 38.10 |
| Rectus_R | 84.46% | 73.68% | 95.24% |
| Rolandic_Oper_L | 58.02% | 68.42% | 47.62% |
| Rolandic_Oper_R | 70.33% | 78.75% | 61.90% |
| Supp_Motor_Area_L | 50.0% | 0% | 100% |
| Supp_Motor_Area_R | 87.59% | 89.47% | 85.71% |
| SupraMarginal_L | 50.0% | 0% | 100% |
| SupraMarginal_R | 50.0% | 0% | 100% |
| Temporal_Inf_L | 67.79% | 73.68% | 61.90% |
| Temporal_Inf_R | 77.57% | 78.95% | 76.19% |
| Temporal_Mid_L | 50.0% | 0% | 100% |
| Temporal_Mid_R | 77.57% | 78.95% | 76.19% |
| Temporal_Pole_Mid_L | 70.43% | 78.95% | 61.90% |
| Temporal_Pole_Mid_R | 65.16% | 68.42% | 61.90% |
| Temporal_Pole_Sup_L | 80.20% | 84.21% | 76.19% |
| Temporal_Pole_Sup_R | 66.54% | 47.37% | 85.71% |
| Temporal_Sup_L | 72.01% | 63.16% | 80.95% |
| Temporal_Sup_R | 69.92% | 58.89% | 80.95% |
| Thalamus_L | 50.0% | 0% | 100% |
| Thalamus_R | 70.43% | 78.95% | 61.90% |
| Cerebelum_3_L | 60.53% | 73.68% | 47.37% |
| Cerebelum_3_R | 64.92% | 63.16% | 66.67% |
| Cerebelum_4_5_L | 61.28% | 36.84% | 85.71% |
| Cerebelum_4_5_R | 68.30% | 84.21% | 52.38% |
| Cerebelum_6_L | 72.81% | 78.95% | 66.67% |
| Cerebelum_6_R | 67.79% | 73.68% | 61.90% |
| Cerebelum_7b_L | 50.0% | 0% | 100% |
| Cerebelum_7b_R | 74.94% | 73.68% | 76.19% |
| Cerebelum_8_L | 55.54% | 63.16% | 47.62% |
| Cerebelum_8_R | 67.04% | 57.89% | 76.19% |
| Cerebelum_9_L | 63.36% | 64.82% | 61.90% |
| Cerebelum_9_R | 77.57% | 78.95% | 76.19% |
| Cerebelum_10_L | 84.96% | 84.21% | 85.71% |
| Cerebelum_10_R | 72.56% | 73.68% | 71.43% |
| Cerebelum_Crus1_L | 55.27% | 63.16% | 47.37% |
| Cerebelum_Crus1_R | 65.16% | 68.42% | 61.90% |
| Cerebelum_Crus2_L | 56.27% | 31.58% | 80.95% |
| Cerebelum_Crus2_R | 77.57% | 78.95% | 76.19% |
| Vermis_1_2 | 75.44% | 84.21% | 66.67% |
| Vermis_3 | 64.66% | 57.89% | 71.43% |
| Vermis_4_5 | 69.05% | 66.67% | 71.43% |
| Vermis_6 | 77.57% | 78.95% | 76.19% |

GFC = global-brain functional connectivity; AAL = Anatomical Automatic Labeling

Table S2. Group differences of functional connectivity in M1-SMA couplings between patients with CD and healthy controls.

| connectivities | Patients (n=19) | | Controls (n=21) | | *T* value | *p* value |
| --- | --- | --- | --- | --- | --- | --- |
|  | Mean | SD | Mean | SD |  |  |
| M1_L-SMA_L | 0.936 | 0.291 | 0.991 | 0.206 | -0.699 | 0.497^a^ |
| M1_L-SMA_R | 0.721 | 0.263 | 0.854 | 0.168 | -1.925 | 0.062 ^a^ |
| M1_R-SMA_L | 0.733 | 0.332 | 0.855 | 0.338 | -1.146 | 0.259 ^a^ |
| M1_R-SMA_R | 0.864 | 0.309 | 1.074 | 0.303 | -2.169 | 0.036 ^a^ |

a The *p* values were obtained by two samples *t*-tests.

CD = cervical dystonia; SMA = supplementary motor area; M1 = primary motor area; L = left; R = right; SD = standard deviation.

**Calculation of FC between M1 and SMA**

We computed the FC between M1 and SMA using the Anatomical Automatic Labeling (AAL) templates of these regions as ROIs in each participant (left M1-left SMA, left M1-right SMA, right M1-left SMA, right M1- right SMA). FC values of these connectivities were extracted by the DPABI software package and analyzed by independent samples *t*-test with the SPSS software. As shown in Table S2, decreased FC between right M1 and right SMA was found in patients with CD compared with healthy controls (*p*=0.036).
